# Conventional therapy for genital herpesvirus and remission of HPV-related lesions: a case series

**DOI:** 10.1186/s13027-023-00511-0

**Published:** 2023-06-02

**Authors:** Maria Balestrieri, Caterina Carnovale-Scalzo, Anna Rosa Garbuglia, Maria Vincenza Chiantore, Luisa Accardi, Paola Di Bonito

**Affiliations:** 1 Gynaecology and Diagnostic Colposcopy Clinic, Via Enea, 23, 00181 Rome, Italy; 2 Histopathology Laboratory, Ospedale San Carlo di Nancy, GVM Care and Research, Via Aurelia 265, 00165 Rome, Italy; 3 Laboratory of Virology, National Institute for Infectious Diseases Lazzaro Spallanzani, IRCCS, Via Portuense 292, 00149 Rome, Italy; 4 Department of Infectious Diseases, EVOR Unit, Istituto Superiore di Sanità, Viale Regina Elena 299, 00161 Rome, Italy

**Keywords:** Acyclovir, Valacyclovir, HHV-2, HPV, Condylomatosis, STI, Cervical lesions

## Abstract

This report covers the case of 7 women affected by pathologies related to genital Herpesvirus and Papillomavirus. They were referred to the gynaecology outpatient clinic for colposcopic examination, and received pharmacological antiviral treatment. The patients presented clinical signs of genital Herpesvirus infections in the cervix and vulva. Cervical lesions and condylomatosis, which are characteristic of Papillomavirus infections were also detected, and patients underwent cervical cancer screening. Patients received oral and topical treatment with Acyclovir or oral treatment with Valacyclovir. During weekly or biweekly gynaecological follow-up visits, patients showed different times of remission of genital Herpesvirus. During the antiviral treatments, the vulvar and cervical Papillomavirus lesions also showed complete resolution with *restitutio ad integrum* of the tissues, and no recurrence at follow-up visits. Herpesvirus and Papillomavirus infections are often associated in genital infections and, as sexual transmitted infections, share the same risk factors. In the cases presented, the observed remission of HPV-related pathologies during Acyclovir and Valaciclovir treatments may suggest that antivirals are also effective in the treatment of HPV lesions. The cases described could pave the way for further investigations and clinical studies.

**Supplementary Information:**

The online version contains supplementary material available at 10.1186/s13027-023-00511-0.

## Main text

### Patient characteristics

**Fig. 1:**
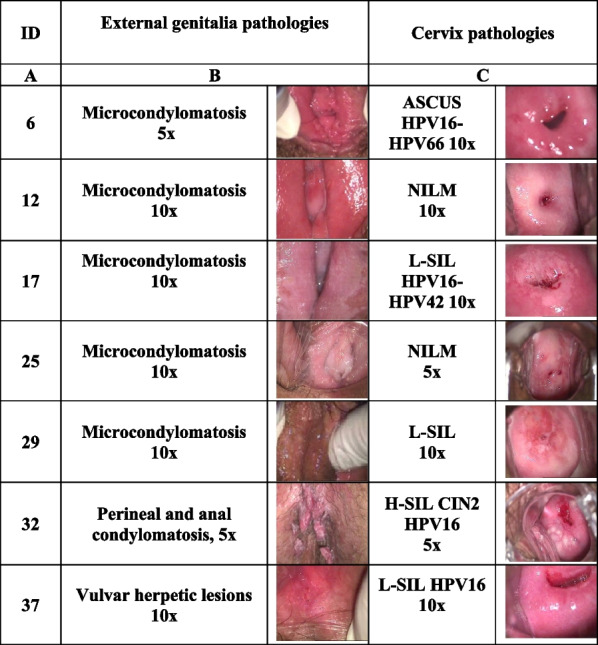
Colposcopy images of genital pathologies of women treated with antivirals. The columns report: **A** ID patient identification number; **B** images of clinical lesions in the external genitalia; **C** images of the cervix and Pap-test results, according to the Bethesda system are also reported for each patient; the magnification (5x, 10x and 20x) of images is indicated. Herpetic lesions are evident in ID6, 25 and 29 cervix and in ID12, 17, 25, 32 and 37 vulva. Colposcopic inspection was performed with the Binocular colposcope OP-C2, OPTOMIC with 5X, 10X and 20X magnifications. Digital images were archived using the Sinet healthnet suite 6.0 program by CSI NET srl

The pathologies described in this report regard women who had been to the outpatient clinic of a specialist in obstetrics and gynaecology. Following colposcopic examination, patients showed clinical signs of genital herpesvirus type 2 (HHV-2) and Papillomavirus (HPV)-related lesions. The patients’ clinical data are summarised in Table 1, and the colposcopic images of the pathologies treated are shown in Fig. 1. Patient ID and age are reported in Table 1, in columns A and B. All patients had vulvar or cervical lesions caused by genital HHV-2 (column C), and underwent cervical cancer screening, including cytology by ThinPrep specimens, HPV testing and typing, and cervical biopsy in one case (ID 32). HPV-DNA test was performed only in case of cytology abnormalities. The HPV-related pathologies detected are listed in column D. Atypical squamous cells of undetermined significance (ASC-US) were observed in ID6 Pap-test, while low-grade squamous intraepithelial lesions (L-SIL) were observed in ID 17, 29 and 37 Pap-tests. Patient ID29’s HPV-DNA test was negative, while the other mentioned patients had HPV-DNA test positive for the high-risk (HR) HPV16. HR-HPV66 and low-risk (LR)-HPV42 genotypes were also detected in cervical specimens from patients ID6 and ID17, respectively. Patient ID32 showed a high-grade intraepithelial lesion which was found to be CIN2 on histological examination after biopsy (column D). Patients ID12 and ID25 showed no cell abnormalities or HPV infection in Pap smear of cervical cells. HPV-related genital condylomatosis was observed in all patients except ID37 (column E), and candidiasis was observed in patients ID6, ID17, ID25, ID29 and ID32 (column F). The colposcopy images of clinical signs (blisters) of HHV-2 in vulva (ID12, 17, 25, 32 and 37) or cervix (ID6, 25 and 29), the perineal and anal condylomatosis (ID32), the vulvar microcondylomatosis (ID6, 12, 17, 25 and 29), and the HPV-related cervical lesions (ID6, 17, 29, 32 and 37) are shown in Fig. 1.

**Table 1:** Clinical data of patients

ID	Age	Genital HHV-2	PAP TEST HPV-DNA	Condylomatosis	Other STI	Oral antiviral dosage	ACV/VCV topical dosage	Times of remission (d, m, y)
A	B	C	D	E	F	G	H	I
6	27	Cervical lesions	ASCUS HPV16-HPV66	Vulvar micro-condylomatosis	Severe candidiasis	VCV 3000 mg/d/90d	No	2 m
12	39	Vulvar lesions	NILM	Vulvar micro-condylomatosis	(Past laser treatment of CIN2)	ACV 1600 mg/d/15d	No, due to vulvodynia	20d
17	27	Vulvar lesions	L-SIL HPV16-HPV42	Vulvar condylomatosis	Candidiasis	VCV 2000 mg/d/30d	5% (2x/d/20d)	2 m
25	40	Vulvar and cervical lesions	NILM	Vulvar micro-condylomatosis	Vulvo-perineal candidiasis	ACV 1600 mg/d/15d	5% (3x/d/10d) 2 times with 7d interval	15d
29	42	Cervical lesions	L-SIL HPV negative	Vulvar micro-condylomatosis	Candidiasis	VCV 1000 mg/d/42d	No	5 m
32	40	Vulvar HHV-2 recurrent	H-SIL CIN2 HPV16	Perineal and anal condylomatosis	Candidiasis	VCV 2000 mg/d/90d	5% (1x/d/45d)	6 m
37	48	Vulvar lesions	LSIL HPV16	nd	nd		5% ACV (intravaginal appl 4x/d/15d) 4 times with 7d interval	7 m

The columns report from left to right in order: A) ID patient identification number, B) age, C) presence of clinical herpesvirus (HHV) lesions, D) PAP and HPV-DNA test results, E) presence of condylomatosis, F) Candida co-infections, G) ACV or VCV oral antiviral dosage, H) topical dosage administered and I) time of remission, indicating the time elapsed from the first visit to the visit in which *the restitutio ad integrum* of the tissue was ascertained. HPV DNA test was not performed in ID12 and ID25 patients, because their Pap-test result did not show cytology abnormalities

*HPV* Human Papillomavirus; *ASC-US* atypical squamous cells of undetermined significance; *NILM* Negative for intra-epithelial lesions and malignancy; *LSIL* Low-grade Squamous intraepithelial lesion; *HSIL* high-grade Squamous intraepithelial lesion; *HHV* Human Herpesvirus; *ACV* Acyclovir; *VCV* Valacyclovir; *d* day/s; *m*: month/s; *y* year/s. *1x; 2x; 3x*:1, 2, 3 times repeated; *Nd* not detected; *Appl* application

### Treatments

Patients were treated with oral and topical standard doses of Acyclovir (ACV), or its derivate Valacyclovir (VCV), which has a higher oral bioavailability [1]. ACV and VCV are the gold standard drugs for the treatment of HHV-1 and HHV-2 infections [2].

For each patient, systemic treatment with ACV or VCV, alone or in combination with topical treatment, was recommended for different periods in relation to the severity of the case and the patient’s health conditions, as deduced from the Summary of Product Characteristics (SPC) in force in Italy. A biweekly gynaecological visit, including a colposcopy examination when necessary, was scheduled as a follow-up of HPV-derived pathologies. Instead, patient ID32 with CIN2 diagnosis received a weekly visit in the first period of therapy. In Table 1, column G, the oral antiviral dosage is indicated for each patient. The topical administration of 5% ACV cream was suggested to the patients with HHV-2 vulvar lesions with the exception of the ID 12 patient who was affected by vulvodynia. In cases of candidiasis, the patients were treated topically with 1-week cycles of 1% Econazole nitrate (Pevaryl). The patients received instructions regarding daily personal hygiene (Additional file 1).

### Observations at follow-up

The specialist offered to patients with multiple infections a gynaecological visit including a colposcopic examination, if necessary, every 1–2 weeks. Through these follow-ups, the specialist was able to balance the drug treatment period and observe disease improvements, including the remission of HPV-related cervical lesions and vulvar condylomatosis. During the antiviral treatment, the follow up gynaecological visits revealed not only the healing of the herpetic lesions, but also the remission of the cervical and vulvar HPV-related lesions.

In HHV-infected cells, for antivirals to function, they must be phosphorylated by viral thymidine kinase (HHV-TK) to inhibit DNA synthesis and viral replication by HHV-DNA polymerase, for which ACV has a high affinity [3]. However, it has been shown that cellular thymidine kinase can replace viral kinases [4], and ACV also showed inhibitory activity against cellular DNA Polymerases [5, 6]. Importantly, an in vitro study showed that the ACV treatment of HPV18-transformed HeLa cell line induced growth arrest, cell proliferation inhibition, and reduced cell survival with the formation of micronuclei [7]. Furthermore, the efficacy of ACV has also been demonstrated by intralesional administration in cutaneous warts [8, 9], as well as by postoperative therapy in Recurrent Respiratory Papillomatosis [10], both HPV-related pathologies. Two clinical trials on the efficacy of ACV against  plantar warts are currently ongoing (NCT05429151; NCT05324904).

## Conclusions

To our knowledge, these are the first clinical observations on the possible efficacy of ACV on HPV-related anogenital lesions. Genital HHV infections have increased enormously in recent years [11], and the possible efficacy of ACV also on HPV-related clinical manifestations could have an impact on the high epidemiological burden of anogenital HPVs in countries where HPV vaccination is not widespread [12, 13].

## Supplementary Information

**Additional file 1**. Instructions to the patient for intimate hygiene.

## Data Availability

Anonymous patient records are available for consultation upon specific request and in compliance with the laws in force in Italy.

## References

[1] Majewska A, Mlynarczyk-Bonikowska B. 40 years after the registration of acyclovir: Do we need new anti-herpetic drugs? Int J Mol Sci. 2022;23(7). 10.3390/ijms23073431 PMC8998721 35408788

[2] WHO. WHO Guidelines for the Treatment of Genital Herpes simplex virus. WHO Libr. 2016;8(4):207–11. 27875039

[3] De Clercq E, Li G (2016). Approved antiviral drugs over the past 50 years. Clin Microbiol Rev.

[4] Chen S-H, Cook WJ, Grove KL, Coen DM (1998). Human thymidine kinase can functionally replace herpes simplex virus type 1 thymidine kinase for viral replication in mouse sensory ganglia and reactivation from latency upon explant. J Virol.

[5] Clair St. MH, Furman PA, Lubbers CM, Elion GB. Inhibition of cellular α and virally induced deoxyribonucleic acid polymerases by the triphosphate of acyclovir. Antimicrob Agents Chemother. 1980;18(5):741–5. 10.1128/aac.18.5.741 PMC284085 7192534

[6] Ilsley DD, Lee SH, Miller WH, Kuchta RD (1995). Acyclic guanosine analogs inhibit DNA polymerases α, δ, and ε with very different potencies and have unique mechanisms of action. Biochemistry.

[7] Jagetia GC, Aruna R (1999). Effect of various concentrations of acyclovir on cell survival and micronuclei induction on cultured HeLa cells. Mutat Res Genet Toxicol Environ Mutagen.

[8] Elsayed A, Nassar A, Marei A, Hoseiny HAM, Alakad R (2022). Intralesional acyclovir: a potential therapeutic option for cutaneous warts. J Cutan Med Surg.

[9] Bagwell A, Loy A, McFarland MS, Tessmer-Neubauer A (2016). Oral acyclovir in the treatment of verruca. J Drugs Dermatol.

[10] Mitra S, Das A, Ghosh D, Sengupta A (2019). Postoperative systemic acyclovir in juvenile-onset recurrent respiratory papillomatosis: the outcome. Ear Nose Throat J.

[11] Looker KJ, Johnston C, Welton NJ, James C, Vickerman P, Turner KME (2020). The global and regional burden of genital ulcer disease due to herpes simplex virus: a natural history modelling study. BMJ Glob Heal.

[12] Kombe Kombe AJ, Li B, Zahid A, Mengist HM, Bounda GA, Zhou Y (2021). Epidemiology and burden of human papillomavirus and related diseases, molecular pathogenesis, and vaccine evaluation. Front Public Health.

[13] Spayne J, Hesketh T. Estimate of global human papillomavirus vaccination coverage: analysis of country-level indicators. BMJ Open. 2021;11(9). 10.1136/bmjopen-2021-052016 PMC8413939 34475188

