# Supplementary material for: Conventional therapy for genital herpesvirus and remission of HPV-related lesions: a case series
Source: Infect Agent Cancer. 2023 Jun 2;18:36. doi: 10.1186/s13027-023-00511-0 (PMC10236747; doi:10.1186/s13027-023-00511-0)
Supplement: Supplementary file 1 — Additional file 1. Instructions to the patient for intimate hygiene. [file 13027_2023_511_MOESM1_ESM.docx]

**Supplementary file**

**S1**. Patients received instructions regarding daily personal hygiene to be followed during the antiviral treatment for a time period depending on the disease (1 or 2 weeks or 10 day cycles).

1) Chlorhexidine 0.2%, soap/detergent and vaginal ova.

2) Vidermina clx mousse, or Vidermina clx vaginal cleansing solution, 5 min local treatment before rinsing with water.

-Vidermina clx vaginal ova.

(Vidermina clx composition: water, glycerine, hydroxyethyl cellulose, laureth-9, chlorhexidine digluconate (0.2%), sodium hyaluronate, lactic acid).

3) Sodium hypochlorite topical, 0.05% or 0.01% recommended for condylomatosis.

**S2**. In the presence of severe candidiasis, the patients were treated topically for 1 week with cycles of 1% Econazole nitrate ova (Pevaryl) or cream and Vidermina CLX, Vaginal Wash Solution, and Marseille soaps for daily personal hygiene. (Pevaryl).
